# The Translational Genomics Core at Partners Personalized Medicine: Facilitating the Transition of Research towards Personalized Medicine

**DOI:** 10.3390/jpm6010010

**Published:** 2016-02-26

**Authors:** Ashley Blau, Alison Brown, Lisa Mahanta, Sami S. Amr

**Affiliations:** 1 Partners HealthCare Personalized Medicine, 65 Landsdowne Street, Cambridge, MA 02139, USA; ablau@partners.org (A.B.); abrown13@partners.org (A.B.); lmahanta@partners.org (L.M.); 2 Department of Pathology, Brigham and Women’s Hospital and Harvard Medical School, Boston, MA 02115, USA

**Keywords:** translational genomics core, academic core facility, genomics services, genotyping, seamless workflows

## Abstract

The Translational Genomics Core (TGC) at Partners Personalized Medicine (PPM) serves as a fee-for-service core laboratory for Partners Healthcare researchers, providing access to technology platforms and analysis pipelines for genomic, transcriptomic, and epigenomic research projects. The interaction of the TGC with various components of PPM provides it with a unique infrastructure that allows for greater IT and bioinformatics opportunities, such as sample tracking and data analysis. The following article describes some of the unique opportunities available to an academic research core operating within PPM, such the ability to develop analysis pipelines with a dedicated bioinformatics team and maintain a flexible Laboratory Information Management System (LIMS) with the support of an internal IT team, as well as the operational challenges encountered to respond to emerging technologies, diverse investigator needs, and high staff turnover. In addition, the implementation and operational role of the TGC in the Partners Biobank genotyping project of over 25,000 samples is presented as an example of core activities working with other components of PPM.

## 1. Introduction

Technological and analytical advancements in genomics and decreasing cost of sequencing and array-based platforms in recent years are a key catalyst in the exponential growth of translational research initiatives and broader integration of clinical genetic testing. Most notably, next-generation sequencing (NGS) technologies have enhanced the capabilities of researchers and clinical laboratories to identify and characterize markers of disease in pursuit of personalized medicine. More recently developed technologies, such as NGS, as well as established platforms, such as array-based technologies, require significant laboratory infrastructure and operational expertise to maximize their utility, and therefore, are often aggregated in core facilities at academic medical institutions. In its commitment to advancing medical genomics research and its integration into clinical practice, Partners Healthcare Personalized Medicine (PPM) established the Translational Genomics Core (TGC) as part of its center to provide access and expertise of leading technologies to academic clinical and translational researchers at Partners Healthcare.

The TGC offers end-to-end technical and analytical expertise and services across several leading platforms that support genomic, transcriptomic and epigenomic studies (Table 1). All platforms and assays performed at the TGC are validated and optimized on automation instruments to provide reproducible, high quality, and high-throughput sample processing and data generation. In addition, the core is responsible for the evaluation and development of novel platforms and assays to further research programs at Partners Healthcare. Alongside its role as a core facility to researchers, the TGC is integrated into the initiatives and operations of other components within Partners Personalized Medicine (described in “An overview of Partners Personalized Medicine at Partners Healthcare” in this issue). As a result of these interactions, the TGC enjoys a synergistic relationship with these different components that have translated into unique opportunities for the center and Partners researchers. The following describes the role and operations of the TGC within the center and across the Partners Healthcare research community.

## 2. Supporting Partners Investigators

**Figure 1:**
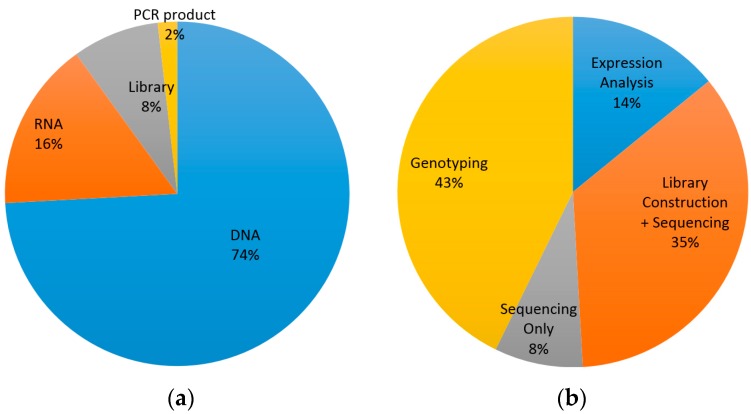
Percentage distribution of samples submitted by (**a**) type of sample submitted and (**b**) processing platform; represents 11,155 samples from 75 investigators submitted over a one year period (September 2014–September 2015).

Over the past year, the Translational Genomics Core has served approximately 75 investigators to help them achieve their research goals through access to genomics platforms and services in Table 1, providing end-to-end processing and analysis of research samples. These services are offered to Partners Healthcare researchers with institutional, grant, or commercial funding on a first-come first-serve basis. More than 11,000 samples were submitted to the TGC for processing across the various platforms offered. Of all samples types submitted, approximately 75% were DNA, and the rest were RNA, PCR product, or pre-made libraries. The majority of samples received for processing were for genotyping and library construction and NGS sequencing services. Of the almost 5000 samples processed on the Infinium platform for either genotyping or methylation this year, about half were for the Partners Biobank on Illumina’s Multi-Ethnic Consortium Genotyping Array (MEGArray) chip. A breakdown of the types of samples submitted for the various services can be viewed below (Figure 1).

**Figure 2:**
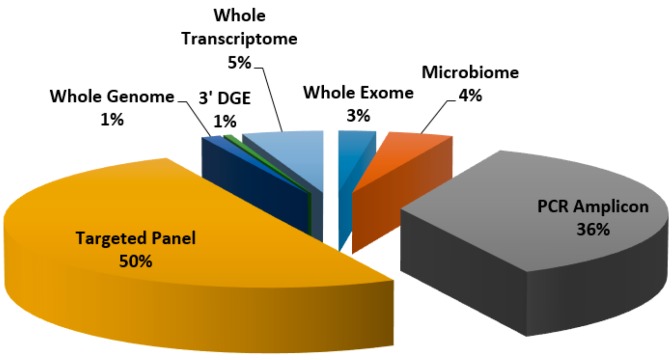
Breakdown of volume of samples submitted for various library construction methods; represents 3441 samples submitted from 20 unique investigators over a one year period (September 2014–September 2015).

The Translational Genomics Core (TGC) pays close attention to researcher focus and technology needs. To make sure researcher needs are met, the TGC is continuously discussing new methods with investigators and companies and developing new technologies in the lab. The disease areas studied by Partners investigators are vast so many different methods and platforms are required to support this diverse range of work. A list of large research areas studied by Partners investigators that have been supported by the TGC can be found in Table 2, and a breakdown of the many library construction services utilized by investigators can be viewed in Figure 2.

## 3. Integration of Core Operations through LIMS

At the foundation of Partners Personalized Medicine is the operations driven by information technology (IT). The same (Laboratory Information Management) LIM systems, which is a software based management system to support any and all laboratory operations, are utilized across departments, allowing for end to end project management. These LIMS and the IT infrastructure are described in depth in “The Information Technology Infrastructure for the Translational Genomics Core and the Partners Biobank at Partners Personalized Medicine” in this issue. For research projects two main LIMS are utilized, STARLIMS and GIGPAD. STARLIMS is used to manage sample processing workflows in the lab. It also serves as a sample inventory tracking system capturing the physical location of samples within the lab. If investigators have their own external samples to submit for processing, they can place orders through GIGPAD (https://portal.hpcgg.org/). Investigators can upload their sample information directly into the portal and order sample processing and analysis services. The genomic instruments and analysis software in the lab can feed output data directly into this LIMS allowing data to be viewed and downloaded securely by investigators through this platform. GIGPAD is also used to bill for rendered services. GIGPAD interfaces with STARLIMS, so updates in the processing workflow made by the lab in STARLIMS are automatically updated in GIGPAD for the investigator to view.

The same IT team supports all departments at Partners Personalized Medicine. One of the roles of the IT team is to manage both GIGPAD and STARLIMS. Having the same team manage both LIMS helps maintain the integrity of data being shared between the two platforms. This unification of support also helps streamline troubleshooting for the ongoing clinical and research processes within Partners Personalized Medicine as well as and external investigators. Members from the lab and IT teams meet weekly to provide updates, feedback on functionality, and bring any issues to discuss. This ongoing dialogue helps keep operations running smoothly.

The Partners Biobank houses banked samples (plasma, serum, and DNA) collected from research-consented patients from Partners Healthcare hospitals, which are available to Partners researchers or distribution to Partners HealthCare investigators with appropriate approval from the Partners Institutional Review board (IRB) (described in “The Partners HealthCare Biobank at Partners Personalized Medicine” article in this issue). Whether samples were obtained through the Partners Biobank or were from an investigator’s private cohort, the Biobank can provide samples processing services, such as extraction, quantitation, and plating. The Biobank is proficient at preparing samples to meet required specifications for various downstream processing platforms offered by the Translational Genomics Core. Many investigators take advantage of this end to end workflow, and will submit samples to the Biobank for preparation to be processed by the TGC. The same lab manager and project manager oversee projects in both the Biobank and the TGC. This integration between departments lends to a smooth transition of projects from sample processing to the genomic analysis stage. The same LIMs and PPM staff track projects from the time the initial request is made, through sample preparation in the Biobank and genomic analysis in the Translational Genomics Core, until the return of data. If analysis is requested, the project will be tracked through the Bioinformatics team until analyzed data is released to the investigator, through a secure File Transfer Protocol (FTP) on the Partners Healthcare network for Partners Healthcare researchers, or through an external hard drive. 

## 4. Providing Bioinformatics (BioFx) Support on Core Areas of Expertise

Processing and analysis of sequencing and genotyping data requires complex bioinformatics tools and pipelines to provide reliable results that can be accurately interpreted in the context of the study. Working closely with the bioinformatics team at PPM, the TGC is able to provide bioinformatics services and expertise to translational and clinical researchers. Given the enormous time and effort required to build bioinformatics tools and gain analytical expertise, the TGC has aligned its bioinformatics development goals and services with that of different components of the center. For example, leveraging the LMM’s expertise in sequencing analysis, “clinical” grade variant calling and comprehensive annotation for whole genome/exome and targeted sequencing projects is offered to researchers through the core. For researchers with limited expertise in the development of bioinformatics tools, this has led to confidence in analytical methods and efficient utilization of genomic sequencing data. Although many bioinformatics requests have been encountered, we have limited our services to focus on those that are more common to investigators at Partners Healthcare

In addition, we have focused our in-house bioinformatics development efforts to support the technology platforms most utilized by the majority of translational and clinical investigators including: (1) variant calling and annotation for targeted sequencing panels, exomes, and whole genomes, (2) differential expression through RNAseq, (3) allele calling on genotyping arrays, and (4) differential expression on microarray platforms. To this end, our strategy is to partner with research groups with a vested interest in a given assay or platform. This allows both groups to share resources and expertise in building an end-to-end solution for novel techniques and platforms. These partnerships often arise with repeat external users of center resources who want to develop a new experiment protocol that requires special equipment and expertise for the wet bench portion and is an area of interest in the research community. We are able to contribute to this partnership by providing the development of the wet bench process and access to the specialized equipment. 

One such example, is the development of a robust whole transcriptome sequencing analysis pipeline through a collaboration between the TGC and a Partners investigator with training and expertise in bioinformatics analysis. Through this interaction, the core provided access and technical expertise in library construction from RNA samples and sequencing of libraries on the NGS platform. The investigator, in turn, developed an analysis pipeline using the Tuxedo Suite tools (Broad Institute) that includes alignment of reads to a reference genome, measures transcript abundance, and performs differential expression analysis and visualization. Through several rounds of validation experiments and analyses, we optimized our wet-bench and analysis workflow to include quality control (QC) measures such as synthetic oligo spike-ins and fastq QC analysis which help inform on the quality of samples and their processing in the lab, and then set “pass” thresholds to avoid the use of inaccurate data. A copy of this pipeline was transferred to PPM servers and is maintained and offered to other researchers through our bioinformatics group, providing a laboratory processing workflow with QC checks at multiple steps and access to a validated transcriptome sequencing analysis pipelines. Similar initiatives to co-develop bioinformatics tools for platforms and techniques that support personalized medicine research are underway, including metagenomics 16S rRNA gene microbial profiling and small RNA/miRNA profiling and discovery from serum and plasma samples.

## 5. Supporting Biobank Genotyping Initiative

A major goal of the Partners Personalized Medicine Biobank is the genotyping of Biobank samples using the MEGArray chip on Illumina’s Infinium platform. To complete objectives of this genotyping initiative, the TGC is required to meet a throughput of 1000–1500 samples per month to be run on this chip. Genotyping hardware and infrastructure already existed in the TGC for genotyping small projects with a low sample number. The increase in volume and throughput required by the Biobank genotyping project meant that we required additional resources, equipment, modification to existing equipment and a sample handling and wet lab processing LIMS. The Illumina Laboratory Information Management System (LIMS) is a proprietary system used to connect sample ID’s in sample plates with all reagent barcodes and to collect technician and instrument ID’s and timestamps for complete documentation of the wet lab process from start to finish. Manual collection of this information with a hand-held barcode scanner was possible but slowed the process dramatically and was liable to introduce error. This made it necessary to integrate a robotic manipulator arm (RoMa) to the Tecan EVo liquid handling system (Tecan) for all the post PCR sample handling and manipulation. The RoMa integrates two barcode scanners necessary for capturing sample, reagent, and chip identity throughout the process. The capacity of our scanner was doubled by the introduction of a second scanner with a twister arm to allow walkaway continuous scanning of chips into the LIMS. These improvements allow processing of 384 samples per week by one technician, with throughput doubling with two technicians on a staggered schedule.

**Figure 3:**
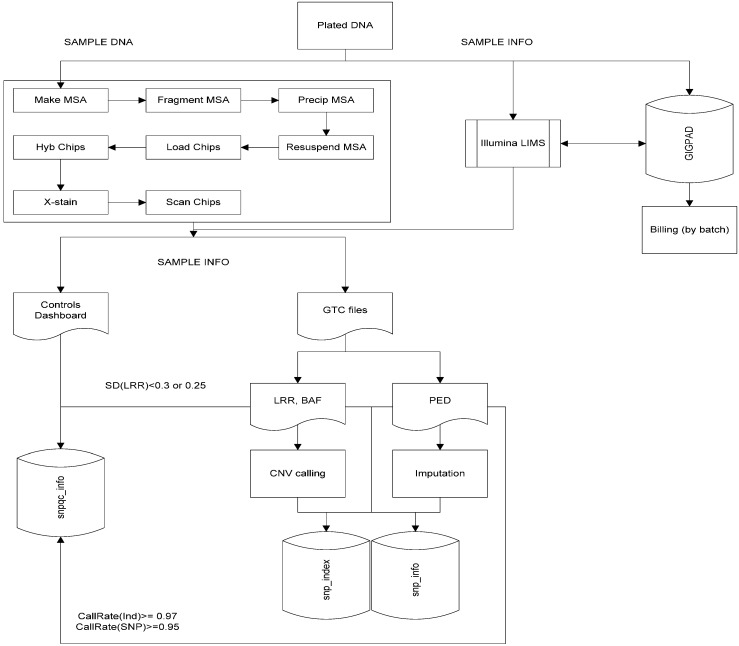
Summary of Biobank genotyping workflow (taken from Tsai *et al.* [1]).

On completion of scanning the chips, data (.idat) is collected in the Illumina LIMS and a previously generated cluster file (.egt file) from Illumina GenomeStudio software is applied to it along with a SNP manifest file specific for the version of the chip used (.bpm). The cluster file assigns an allele call to each sample per SNP based upon the intensity of the fluorescent dye found at a position on the chip during scanning. The ID of the SNP is based on the position of the call on the chip using the manifest (.bpm) as a reference. This produces the genotype call files (GTC) that are used to create smaller binary PED files. PEDs from each sample are merged together and then curated to remove failing SNPs, which are SNPs having a call rate <0.95% across all samples, or SNPs that are not mapped to the genome. At this point the Biobank IDs are re-associated with the data as well as a gender designation. The genotyped gender is compared to the assigned gender as a QC check. Quality control plots are generated to allow identification of failing samples; those that are lower than 97% success across all targeted positions or show an intensity signal lower than the intensity of the background noise. An overview of the genotyping workflow is shown in Figure 3 which highlights the sample wet bench processing, integration of the Illumina LIMS and our homebrew GIGPAD LIMS, as well as initial bioinformatics analyses that offer basic genotyping calls and quality control check based on SNP intensities and/or call rates. Additional information on the bioinformatics workflow is described in Tsai *et al*. [1]. For the TGC, the utility of this integration between the LIMS and Biofx processes is in its ability to perform cluster file generation using multiple batches run on the same array, thereby enhancing allele calling, particularly to rare SNPs, and lower the SNP failure rate. In addition, the generation of sample QC plots for multiple batches simultaneously, which occurs at the “snpqc_info” step in Figure 3 and is described in Tsai *et al.* [1], allows the laboratory team to more easily identify failed samples and batch specific trends relating to sample quality or chip lot quality due to manufacturer error. 

## 6. Staffing and Operations Challenges at an Academic Core

The field of genomics is a rapidly changing field with the introduction of new techniques and technologies on a regular basis allowing genomics to be carried out faster and cheaper than previous iterations. Generally, core labs are forced by budgetary constraints to recruit inexperienced graduates and train them on the job. Once hired, retention then becomes an issue, as technical staff with growing experience have the option of moving to higher paying similar roles in surrounding industry labs. Balancing staffing levels with demand is tricky as volume of incoming work is highly variable with little long term planning and communication between the researcher and the core lab. High turnaround of staff and constantly changing services requires close management to ensure the laboratory remains in GLP (Good Laboratory Practice) compliance [2]. This is an important quality measure for our customers, especially those who may be working towards FDA approval as part of their research. While success of a core facility can be measured by financial success, in an academic environment another measure of success is through publication, either directly by the core facility or through recognition of the core within publications by our customers, or the number of grants that a core facility supports. Following below is a more detailed discussion on how we deal with each of these challenges with the aim of moving forward within the world of genomics and providing a high quality cost effective service to our customers.

Our facility’s pool of customers includes a large number of translational and clinical researchers whose needs are wide ranging in the field of genomics. As well as carrying out internal review of new technologies and techniques ourselves, we are often approached by customers with novel methods or instrumentation needs, looking for a service lab to support them. The effort required to bring on a new technique is high, so detailed consideration may be required. Technique development demands resources be redirected from income generating production work for a period of time while manual testing, optimization, automation of high volume tests, quality control, procedure documentation and analysis of data is carried out. Other points to consider include availability of staff to carry out development, acceptable timeline for development of the test for the requesting customer, the level of demand for a new test, whether proposed cost to customer will be acceptable and if there are cheaper alternative services available to researchers in other institutional facilities. Introduction of new instrumentation is also high risk, due to the potentially high purchase price. Caution may be warranted to allow time to monitor how well that technology performs in other facilities. If possible, alternative systems should be reviewed to ensure the correct decision is made. Proof of concept testing is required, either with the vendor of the instrumentation or with a known collaborator/colleague. One way for a core facility to mitigate the burden of the instrument purchase price is to partner with a PI or consortium who hold a grant to cover the purchase of a high value instrument and offer to house and operate the instrumentation for a reduced fee, while allowing other customers’ samples to be run on that instrument in downtime. At the opposite end of the spectrum, the decision to stop offering a technique or use of piece of instrumentation, sometimes when there is still low demand for it, is made to offset unnecessary expenditure of resources, labor, and costs associated with maintaining that platform or process. 

The largest three items in our annual budget are supplies, maintenance contracts and salaries. Supply costs can be negotiated with vendors for best prices, but they also vary with volume. The costs of supplies are covered directly by charges to our customers. Maintenance contracts are more static but can also be negotiated with cheaper contracts arising by locking down coverage over multiple years. Salaries are defined on an institutional level depending on qualifications and experience to ensure parity over staff at a particular level within the institution. This makes it difficult to compete in salary for outstanding new hires. Our current staff includes a Director, a Laboratory Manager, a Project Manager and five junior staff. All junior staff are hired with no experience and go through lengthy training on the job, so in order to make this worthwhile we ask for two years as a minimum commitment. There can be hesitancy in accepting a position within a core facility as there is an incorrect perception of the scope of work carried out by junior staff in a fee-for–service lab. Prior to working in our lab, the perception is that it would be mundane, boring and repetitive, but in reality all staff are cross trained on all our services through genotyping, next-generation sequencing and gene expression and so are kept busy and challenged. In addition, during the interviewing process we highlight the broad range of cutting edge clinical and translational research that we are involved in to off-set the negative perception of working in a “core” laboratory. This has attracted junior technicians who are exploring career paths or considering graduate or professional education, who felt the scope and breadth of work done at the TGC will be helpful for their respective goals. However, because of the cutting edge nature of the techniques we use, we do find we have an issue with staff retention. The experience gained by our junior staff is sought after by many of the surrounding private institutions and we find that we lose staff after two years due to more competitive salaries offered by those institutions. We aim to attract high quality junior staff and prevent loss of exceptional staff by promotion based on ability, and by giving ownership of interesting and varied projects. This allows them to gain skills in project design, customer service, time management, research and development, as well as management of more junior staff. We also allow time for the staff to take advantage of the professional development training classes offered by our institution. Another thing that we promote is to have our senior management engage with our junior staff and provide recognition for good quality work. We hope that these benefits provided to our staff are giving them experience and education that will hold them in good stead to be successful as they move into their next role, whether that is in our lab or out of it.

The aim of Good Laboratory Practice (GLP) of the food and drug administration (FDA) is to provide consistently high quality technical work and data over time and over different technical staff. There are many areas covered by GLP including equipment management, training records, standard operating procedures, materials quality, data management and quality processes as detailed in 21 CFR 58 [2]. The challenge for any lab is the vast amount of organization and documentation to establish and maintain GLP. It requires buy in and input from all members of staff, so they must all receive the necessary GLP training. It is important that all staff see how following GLP regulations can improve the quality of data we generate for our customers. The biggest challenge we face is the amount of time we can dedicate to the implementation of GLP. Due to our small staff size and the large range of available services, we have limited resources to devote to this as the bulk of our effort is put towards the production of data to our customers. We have initiated GLP compliance in our lab for certain core services such as next generation sequencing and Infinium array genotyping, and are in the process of working through each of our services and understand that this process may take several years. A published study into University GLP compliance showed that the average years to compliance was four years, ranging from 10 months to seven years [3]. This study identifies several challenges to GLP compliance related to the management of GLP programs, quality assurance training and implementation for technicians, and inadequate documentation of all processes within a GLP workflow [3]. 

One of the measures of success and progress of an academic lab is the production of journal articles. This measure is also true in the case of a Core facility; we monitor publications from our institution on a regular basis to aid in measuring the usage of our facility. In the past, it has been common to find that our facility has not been acknowledged, so we remind our customers on data delivery that an acknowledgement is very much appreciated. This process is becoming more common amongst Core facilities and has been recognized as an issue at several national conferences (NERG and ABRF) and highlighted in an editorial in BioTechniques [4]. However, an alternative measure of success has been to identify the number of grants or grant dollars supported by our laboratories. Our figures for the 2014–2015 academic year show that we have supported over 60 grants totaling over $165 million.

## 7. Conclusion

Our operational experiences highlight common considerations encountered at academic research core such as evolving with the advancement of genomic and transcriptomic technologies and assays, and responding to the diverse needs of investigators at an academic and clinical research institution. As highlighted in this article, several key components have allowed us to thrive in this environment, including: (1) building a core set of genomics and bioinformatics services developed through partnerships and collaborations with a clinical lab or researchers within the institution, (2) having a broad and flexible LIMS system that can handle the tracking and management of variety of sample and library types and can easily adapt and integrate new technologies, and (3) cross training of junior staff across multiple platforms and techniques to engage current staff and limit the impact of staff turnover on productivity. As many academic research cores are fee-for-service with limited funds for development and validation of new workflows and implementing regulatory compliance for current ones, the decision to pursue these initiatives must be weighed against the impact it will have on current production and productivity, and the perceived added benefit of that endeavor. These will not always be clear to research cores, which emphasizes the importance of building relationships with clinical and research labs within the institution. 

The integrative nature of all the components of Partners Personalized Medicine situates the Translational Genomics Core in a unique position among academic core laboratories. This is reinforced by a seamless integration of IT infrastructure and bioinformatics pipelines to allow easy communication, management and tracking of samples and data for both external researchers and PPM research initiatives. The Biobank genotyping initiative, which involves Partners Biobank samples, LIMS infrastructure supporting both the Biobank and the TGC, as well bioinformatics pipelines for quality check steps and analysis, highlights the seamless integration of different components for an efficient workflow at the TGC. As such, the TGC is able to utilize its interactions within the center to offer end-to-end genomic analyses services to academic and clinical researchers, while ensuring that onboarding of novel platforms and assays can rapidly occur to help meet the goals of the center.

## Figures and Tables

**Table 1:** Platforms and services at the Translational Genomics Core.

**Technology Platforms**	
Next Generation Sequencing (NGS)	Illumina HiSeq 2500 instrument Illumina MiSeq instrument
Array-based platforms	Illumina iScan System (Infinium BeadChip) Affymetrix GeneChip Scanner
**Services**	
Genomic analysis	Next generation sequencing: Whole genome sequencing (WGS) Whole exome sequencing (WES) Targeted gene panel sequencing (hybrid capture method or amplicon enrichment method) Metagenomic analysis: 16S rRNA gene microbial profiling
	Array based genotyping: Standard or custom arrays (500,000–5,000,000 SNPs)
Transcriptome analysis	Next generation sequencing: Whole transcriptome sequencing (RNAseq) 3’ differential gene expression (DGEseq) Small RNA and miRNA analysis (Small RNAseq)
	Array based expression profiling Whole transcript expression and analysis 3’ expression profiling (polyA transcript profiling) miRNA profiling
Methylation analysis	Next generation sequencing: Reduced Representation Bisulfite Sequencing (RRBS) Whole-Genome Bisulfite Sequencing (WGBS) Targeted Bisulfite Sequencing
	Array-based methylation analysis Bisulfite converted CpG locus genotyping (~485,000 CpG targets)

**Table 2:** Representative list of 10 disease areas and –omics platforms for translational/clinical research projects studied by 75 Partners investigators over the past year illustrating broad range of clinical research activities supported by the TGC.

Disease Areas	Platform
Asthma	Expression Analysis, Methlyation
Chronic obstructive pulmonary disease (COPD)	RNASeq
Rheumatoid arthritis (RA)	Targeted Gene Panel
Thoracic Cancer	Exome Sequencing, Expression Analysis
HIV Integration	Amplicon Sequencing
Coronary artery disease (CHD)	Exome Sequencing
Parkinson’s/Alzheimer’s Disease	Amplicon Sequencing
Myopathies	Targeted Gene Panel
Hypohidrotic ectodermal dysplasia	3’DGE RNASeq
Diet effects on gut microflora	16S Microbiome rRNA Sequencing

## References

[1] Tsai E.A., Peirce J., Embree K., Brown A., Boutin N.T., Amr S.S., Weiss S.T., Lebo M.S. Bioinformatics Infrastructure for the Partners Biobank Initiative. Poster session presented at the meeting of the American Society of Human Genetics.

[2] CFR—Code of Federal Regulations Title 21 of the FDA (updated August 2015). Good Laboratory Practice for Nonclinical Laboratory. http://www.accessdata.fda.gov/scripts/cdrh/cfdocs/cfcfr/CFRsearch.cfm?CFRPart=58.

[3] Hancock S. (2002). Meeting the Challenges of Implementing Good Laboratory Practices compliance in a university setting. Qual. Assur. J..

[4] (2014). Letter from the Editor. A lack of attribution. BioTechniques.

